# A long-term study of the effects of antiviral therapy on survival of patients with HBV-associated hepatocellular carcinoma (HCC) following local tumor ablation

**DOI:** 10.1002/cam4.197

**Published:** 2014-02-12

**Authors:** Hie-Won Hann, Robert Coben, Daniel Brown, Laurence Needleman, Ernest Rosato, Albert Min, Richard S Hann, Kyong Bin Park, Stephen Dunn, Anthony J DiMarino

**Affiliations:** 1 Liver Disease Prevention Center, Jefferson Medical College, Thomas Jefferson University HospitalPhiladelphia, Pennsylvania; 2 Division of Gastroenterology and Hepatology, Jefferson Medical College, Thomas Jefferson University HospitalPhiladelphia, Pennsylvania; 3 Department of Radiology, Jefferson Medical College, Thomas Jefferson University HospitalPhiladelphia, Pennsylvania; 4 Department of Surgery, Jefferson Medical College, Thomas Jefferson University HospitalPhiladelphia, Pennsylvania; 5 Division of Gastroenterology, Beth Israel Hospital, Albert Einstein University School of MedicineNew York, New York; 6 Department of Internal Medicine, Jefferson Medical College, Thomas Jefferson University HospitalPhiladelphia, Pennsylvania; 7 Kimmel Cancer Center, Jefferson Medical College, Thomas Jefferson University HospitalPhiladelphia, Pennsylvania

**Keywords:** Antiviral therapy and survival, hepatitis B, hepatocellular carcinoma, tumor ablation

## Abstract

The ultimate goal of antiviral therapy for chronic hepatitis B (CHB) is prevention of hepatocellular carcinoma (HCC). Earlier we reported favorable effects of antiviral therapy on survival of HCC patients following curative tumor ablation (Int J Cancer online 14 April 2010; doi: 10.1002/ijc.25382). It was the first observation made in the United States. We now report 12 year follow-up of this patient group. CHB patients with no prior antiviral therapy with a single HCC (≤7 cm) were studied. All patients underwent local tumor ablation as their first option. Patients diagnosed before 1999 received no antiviral treatment while those diagnosed after 1999 received antiviral treatment. Survival between the treated and untreated groups was compared. Among 555 HCC patients seen at our clinic between 1991 and 2013, 25 subjects were eligible. Nine subjects (all male patients, median age 53 years [46–66]) did not receive antiviral therapy while 16 (14 male patients, median age 56 years [20–73]) received treatment. Between the two groups, there was no difference in their median tumor size and levels of alpha-fetoprotein and albumin. However, the survival was significantly different (*P* = 0.001): the median survival of the untreated was 16 months (3–36 months) while that of the treated was 80 months (15–152 months). Fourteen of 16 treated patients are alive to date with two longest survivors alive for ≥151 months. In conclusion, concomitant antiviral therapy for CHB patients with HCC reduces and prevents new/recurrent tumor and improves survival. This novel treatment strategy offers an alternative to liver transplantation in patients with HBV-associated HCC.

## Introduction

The ultimate goal of anti-HBV treatment for patients with chronic hepatitis B (CHB) is prevention of hepatocellular carcinoma (HCC) as shown by prospective 1 and retrospective 2,3 studies of a large number of CHB patients with advanced liver disease.

The same goal can also be applied for prevention of new and/or recurrent HCC in patients who had already developed HCC. Since 2005, we in the United States and researchers in Japan have reported that concomitant antiviral therapy with local tumor ablation reduced the new/recurrence of HCC in patients whose initial HCC was successfully ablated 4–8. We reported our finding online in 2010 (Hann et al., Int J Cancer published online 14 April 2010; doi: 10.1002/ijc.25382) 8. Ours was the first observation made from the patients in the United States. Studies of large cohorts from Hong Kong in 2011 9 and Taiwan in 2012 10 have further confirmed the effect of antiviral therapy on recurrence of HCC after surgery.

Currently, the treatment of choice for patients with small HCC in the United States is liver transplantation. However, shortage of organ supply limits this option for a number of these patients. Here, we present our long-term follow-up results of patients with a single HCC (<7 cm) treated with tumor ablation with or without concomitant antiviral therapy. The sample size is limited but our observation spans from 1991 to present, May 2013.

## Materials and Methods

### Patients

From 1991 to 2013, 555 HCC patients referred to our tertiary Liver Disease Prevention Center, Division of Gastroenterology and Hepatology. Among this group, 25 presented with a single tumor <7 cm in diameter and had not received antiviral therapy prior to HCC diagnosis. The rest of patients with HCC presented with a large, multifocal tumor(s) or with metastasis.

These eligible 25 patients with a single tumor had CHB for years but none of them received antiviral drug prior to HCC diagnosis due to either unavailability of the drug or other reasons. Nine HCC patients were identified between 1991 and 1999. They did not receive antiviral therapy due to unavailability of the drug (designated “the untreated”). From year 2000 to present, 16 patients were eligible for study. They were started on antiviral therapy immediately after diagnosis of HCC (designated “the treated”). We have observed their clinical course for the past 22 years (1991 to May 2013) with a follow-up magnetic resonance imaging (MRI) at 1 month following tumor ablation, subsequently at three monthly intervals and later every 6 months throughout. Various HCC treatment options including liver transplantation were presented and discussed. All patients chose the local tumor ablation as the first option.

### Diagnosis of HCC

After referral, the diagnosis of HCC was reconfirmed at Jefferson by imaging studies including MRI in addition to alpha-fetoprotein (AFP) or histology (biopsy brought from outside). Dynamic MRI showing arterial enhancement with washout was used for final diagnosis of HCC as defined by American Association for Study of Liver Disease guideline 11–13 and by our institution 14 that showed arterial enhancement followed by wash out in the last phase during hepatic arteriography. If MRI evaluation was confirmative, further needle tumor biopsy was avoided for fear of needle track seeding.

### Treatment of HCC

Before treatment, various therapeutic procedures including liver transplantation were discussed with patients. All patients in this study opted for local tumor ablation as an initial treatment.

All patients with a single HCC (≤7 cm) lesion underwent local tumor ablative procedures including surgical resection, transarterial chemoembolization (TACE), radiofrequency ablation (RFA), percutaneous ethanol injection (PCEI), cryoablation, Yttrium90 radioembolization with glass microspheres, or laparoscopic RFA (Lap-RFA). Tumor elimination was considered successful when previously identified contrast enhancement was no longer identified. Follow-up MRI was obtained 1 month later and at 3-month intervals subsequently.

### Antiviral therapy (only oral nucleos(t)ides applied)

Regarding antiviral therapy, lamivudine (LAM) was the only drug available until 2002 when adefovir dipivoxil (ADV) was approved. More drugs were used when they became available; Entecavir (ETV) in 2005, telbivudine (TLV) in 2006, and tenofovir disoproxil fumarate (TDF) in 2008. ADV or TDF was added when patients showed virologic breakthrough (one log increase in HBV DNA level [copies/mL] after reaching the nadir while on LAM). Furthermore, with the increasing preference of combination therapy to monotherapy, TDF or ADV was added to LAM even without virologic breakthrough as time went on. TLV and ETV were also used as anti-HBV drugs for later arrivals. These patients continued on anti-HBV therapy without interruption following the initial diagnosis of HCC.

### HBV DNA quantitation

For HBV DNA levels, a solution hybridization assay (Abbott Laboratories, North Chicago, IL) was used between 2000 and 2002 with a lower limit of detection (LLOD) of 1.6 pg/mL. These values were converted to copies/mL by determining 283,000 copies/mL, per 1 pg/mL of HBV DNA. From 2003 onward, HBV DNA was measured by RT-PCR (polymerase chain reaction) (Quest Diagnostics, Horsham, PA) with a LLOD of 500 copies/mL. Serial dilutions were performed for samples exceeding 5.3 log_10_ copies/mL. Values below this cutoff were assigned a value of 1 log_10_ copies/mL. Recently, the cutoff level is <160 copies/mL by the same laboratories.

### Statistical analysis

To examine the difference between the treated and untreated group in survival and other factors including the age, tumor size, serum albumin, platelets, and HBV DNA level, Mann–Whitney test was used for continuous variables and Fisher's exact for categorical variable. Kaplan–Meier was used for the survival analysis. The study was approved by the Institutional Review Board of Thomas Jefferson University.

## Results

Among the 555 patients who presented with HCC at Liver Disease Prevention Center, Thomas Jefferson University Hospital between 1991 and 2013, all but one were Asian Americans with CHB with or without cirrhosis.

Of those, 25 with a single HCC ≤7 cm in diameter were included in the study. They were Asian American patients and had never received antiviral therapy prior to diagnosis of HCC.

Nine untreated patients were all men with a median age of 53 years (48–66 years). Of the 16 anti-HBV treated patients, 14 were men and two were women with a median age of 57 years (20–73 years). All were HBsAg positive and negative for anti-HCV at the time of initial HCC diagnosis. The majority (7/9 untreated and 13/16 treated) were negative for HBeAg and positive for anti-HBe.

There were no significant differences between the untreated and the treated in the following factors: median tumor size, baseline HBV DNA level, AFP, albumin, and platelet count as shown in Table 1.

**Table 1:** Comparison of the untreated and the treated.

	Untreated (9)	Treated (16)	*P* value (by Mann–Whitney)
Year of diagnosis	1991–1999	2000–2010	
Age	53 (46–60)	57 (20–73)	0.61
Size of tumor (cm)	3 (1–6.9)	2.65 (1–7)	0.67
AFP (ng/mL)	484 (3.1–1864)	19.7 (3–14,101)	0.53
Child-pugh	5A, 2B, 2C	14A, 1B, 1C	0.169 (Fisher's exact)
HBV DNA	8.4 × 10^6^ copies/mL (770–1.6 × 10^7^ copies/mL)	2.4 × 10^5^ copies/mL (500–2.4 × 10^8^ copies/mL)	0.142
Albumin	4.2 (3.1–4.7)	4.2 (3.1–4.9)	0.30
Platelets	126 (62–152)	141 (63–253)	0.12

AFP, alpha-fetoprotein.

### Antiviral therapy

As shown in Table 2, the majority received LAM as the first-line therapy in early 2000 and later TLV in two patients. ADV or TDF were later added. With anti-HBV therapy, HBV DNA became undetectable in all nine patients at median 8 months (range 3–13 months). Three patients developed virological breakthrough with LAM at months 22, 24 and 27, respectively, and were placed on combination therapy with TDF or ADV. In two patients, TDF was added to LAM before virological breakthrough. For those with low baseline HBV DNA, one drug was started and as long as they remained with undetectable HBV DNA, the medication was not changed. All patients on antiviral therapy have maintained undetectable HBV DNA levels and were followed at three to four monthly intervals. All patients underwent curative tumor ablation and the treated group received anti-HBV therapy soon after the index HCC diagnosis was made. Detailed medication and tumor ablation are shown in Tables 3 and 4.

**Table 2:** HBV DNA response on antiviral therapy in the treated group.

Pts	Year Dx	Baseline HBV DNA (copies/mL)	Anti-HBV therapy	HBV DNA (-) in months on anti-HBV therapy
1	2000	3.40 × 10^6^	LAM + TDF later	3
2	2000	6.70 × 10^5^	LAM + TDF later	8
3	2001	1.10 × 10^6^	LAM + TDF later	5
4	2003	8.90 × 10^2^	LAM + ADV later	10
5	2003	5.00 × 10^2^	LAM + TDF later	6
6	2003	7.60 × 10^6^	LAM + TDF later	9
7	2004	5.00 × 10^2^	LAM	3
8	2004	3.30 × 10^5^	LAM + TDF	3
9	2005	1.10 × 10^4^	LMM + ADV	5
10	2006	1.00 × 10^7^	LAM + TDF	8
11	2007	1.30 × 10^5^	TLV + TDF	3
12	2008	4.40 × 10^2^	LAM + TDF	1
13^1^	2008	2.40 × 10^8^	LAM + ADV	4
14	2009	1.40 × 10^7^	TLV + TDF	7
15	2009	1.20 × 10^4^	TDF	6
16	2010	1.40 × 10^5^	TDF	5

LAM, lamivudine; TDF, tenofovir disoproxil fumarate; ADV, adefovir dipivoxil; TLV, telbivudine; HCC, hepatocellular carcinoma.

1 This patient discontinued antiviral drugs 1 year after HCC ablation. One year later (off medicine for a year), he returned with recurrent tumor and HBV DNA 2E + 09 (2 × 10^9^ copies/mL). He died of HCC at 50 months after diagnosis.

**Table 3:** Recurrence of HCC in patients who did not receive antiviral therapy.

Pt	Year of Dx	Age	Sex	Tumor size (cm), site	Child-pugh class	Initial tumor ablation	Antiviral therapy	Recurrence (months)	Retreatment	Survival (months)	Status
1	1991	66	M	2 Rt	C	TACE	None	2	TACE	3	Dead
2	1994	58	M	3 Rt	A	Resection	None	20	Resectionx2	36	Dead
3	1996	46	M	4 Lt	A	Cryoablation	None	5	Cryo, TACE	17	Dead
4	1996	53	M	3 Rt	A	Cryoablation	None	5	TACE, chemo	9	Dead
5	1996	53	M	4 Rt	A	Resection	None	12	TACEx2	16	Dead
6	1997	59	M	1 Rt	B	PCEI	None	2	ChemoRx	6	Dead
7	1999	48	M	2.5 Rt	C	TACE	None	3	TACE multiple	16	Dead
8	1999	60	M	2.5 Lt	A	TACE, RFA	None	9	TACE	17	Dead
9	1999	53	M	6.9 Rt	B	TACE	None	6	Incomplete Ablation, multiple times	6	Dead

HCC, hepatocellular carcinoma; TACE, transarterial chemoembolization; PCEI, percutaneous ethanol injection; RFA, radiofrequency ablation.

**Table 4:** Reduced HCC recurrence in patients on antiviral therapy.

Pts	Year Dx	Age	Sex	Tumor size (cm)	Child-pugh class	Initial tumor ablation	Anti-HBV therapy	Recurrence in months	Retreatment	Survival in months	Status
1	2000	57	M	2.5 Rt	A	RFA	LAM + TDF	None	None	152	Alive
2	2000	64	F	2.5 Rt	A	RFA	LAM + TDF	Residual	TACE	151	Alive
3	2001	49	M	4.0 Rt	A	Resection	LAM + TDF	None	None	140	Alive
4	2003	59	M	1.7 Rt	A	PCEI	LAM + ADV	None^1^	None	116	Alive
5	2003	50	M	2.5 Rt	B	Resection + RFA	LAM + TDF	None	None	116	Alive
6	2003	41	M	2.0 Rt	A	RFA	LAM + TDF	None	None	116	Alive
7	2004	60	M	3.4 Rt	A	TACE + RFA	LAM	None	None	104	Alive
8	2004	55	M	4.6 Rt	A	TACE	LAM + TDF	5 × 4 cm at 6 months	Lap-RFA chemo	15	Dead
9	2005	57	M	3.2 Rt	A	RFA, resection	LAM + ADV	None	None	94	Alive
10	2006	67	M	1.0 Rt	A	RFA	LAM + TDF	None	None	88	Alive
11	2007	20	F	2.8 Rt	A	RFA + TACE	TLV + TDF	None	None	65	Alive
12	2008	73	M	7.0 Rt	B	TACE x2, PCEI, RFA	LAM + TDF	Residual	None	57	Alive
13^1^	2008	65	M	2.3 Rt	A	TACE	LAM + ADV	None	None	50	Dead
14	2009	51	M	4.0 Rt	A	TACE	TLV + TDF	None	None	50	Alive
15	2009	53	M	3.0 Rt	A	TACEx2	TDF	None	None	38	Alive
16	2010	66	M	1.4 Rt	A	TACE	TDF	None	None	26	Alive

HCC, hepatocellular carcinoma; RFA, radiofrequency ablation; LAM, lamivudine; TDF, tenofovir disoproxil fumarate; PCEI, percutaneous ethanol injection; ADV, adefovir dipivoxil; TACE, transarterial chemoembolization; Lap, laparoscopic; TLV, telbivudine.

1 Pt (no. 4) following successful PCEI ablation of Rt lobe HCC remained tumor-free for 7 years. During his trip abroad, he ran out of medicine for 3 weeks. Restarted antiviral drugs. Three months later, on a follow-up visit MRI, a new 6 mm lesion was detected at different site (Lt lobe) of the liver. At 3-month follow-up, the lesion grew to 1 cm and it was TACE'd. Pt (no. 4) is tumor-free for 4 years since and is on antiviral therapy.

### Tumor recurrence and survival

#### Recurrence

As shown in Table 3, all untreated patients developed recurrent or new tumor(s) following the initial tumor ablation with progression at 2–12 months in 8/9 patients. These eight patients died within 17 months. One patient developed new HCC at 20 months and died of metastasis at 36 months.

On the other hand, among the 16 who received antiviral therapy, 14 patients are alive and have continued anti-HBV treatment as shown in Table 4. These 14 received one or repeated ablative procedures with successful results. One 73 year-old man (Pt. no. 12) with a single 7 cm HCC received 2x TACE, PCEI (incomplete due to coughing during procedure) and Lap-RFA for the large HCC in the Rt lobe. A new small lesion in the Lt lobe appeared at 13th month and it was chemoembolized with success. He is alive at 59 months and on antiviral therapy without evidence of viable tumor. One patient (Pt. no. 4), with successful PCEI ablation of Rt lobe HCC and concomitant antiviral therapy, had remained tumor-free for 7 years. During his trip abroad, he ran out of medicine for 3 weeks due to unexpected delay of return. On return home, he immediately restarted antiviral drugs. Three months later, on a follow-up MRI, a new 6 mm lesion was detected at different site (Lt lobe) of the liver. During another 3-month follow-up, the lesion grew to 1 cm and he underwent TACE. He has remained free of recurrence for 4 years since and is on antiviral therapy. Of the two longest survivors who were diagnosed in 2000, one received RFA once and the other RFA followed by TACE. Both are alive without recurrence for over 12 years (152 and 151 months, respectively).

Of the treated patients, two died: One 55-year-old man (Pt. no. 8) developed recurrent tumor at 6 months following initial TACE and died of advanced HCC at 15 months. The other (Pt. no. 13), a 65-year-old man, after successful tumor ablation and with no recurrence, lost follow-up 1 year later. After 1 year during which time he did not receive antiviral therapy, patient returned with recurrent HCC and increased HBV DNA (2 × 10^9^ copies/mL). He died of multifocal HCC at 50 months.

#### Survival

**Figure 1:**
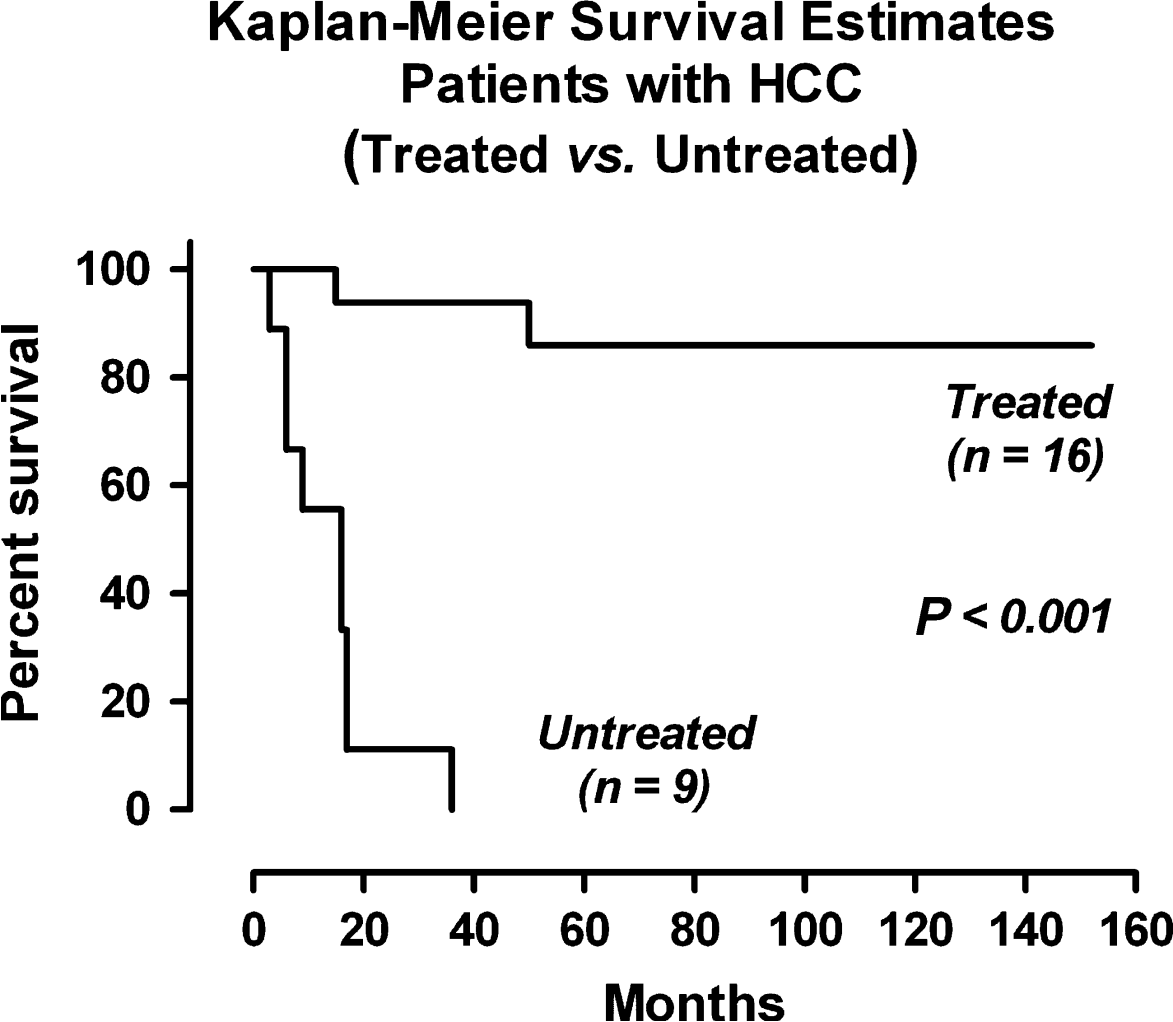
Survival difference between the anti-HBV treated and untreated patients.

The median survival of the untreated was 16 months (ranges 3–36 months) and of the treated was 80 months (15–152 months). The survival difference between the two groups was significantly different (*P* = 0.001) (Fig. 1).

## Discussion

As summarized extensively by Forner et al. 11, patients with HBV-HCC, even after successful treatment of the initial HCC, used to succumb to cancer following multiple recurrences or metastases. Most plausible cause for this outcome is that the hepatocarcinogen, the virus, continues to replicate in the liver and thus the carcinogenic process persists. Following the first introduction of antiviral drug, LAM, it appeared possible that antiviral therapy could reduce the incidence of HCC attributed to suppression of the virus 1–3. Furthermore, in CHB patients with HCC, Piao et al. [4.] and Kuzuya et al. 5 in a median follow-up study of 12 months, reported a favorable effect of anti-HBV (LAM) therapy in reducing tumor recurrence and improving survival following curative resection. Kim et al. 7 observed HCC recurrence rate of 77% in 43 antiviral treated and 92% in 36 untreated patients after a median follow-up of 12 months. These authors attributed the longer survival of anti-HBV treated HCC patients to improvement of liver function with viral suppression and advocated antiviral therapy. In a study of 40 CHB patients with HCC, Kubo et al. 6 concluded that high viral load was one of the risk factors for HCC recurrence following surgical resection. Chan et al. 9 presented 136 patients who underwent major hepatectomy for the HBV-related HCC by one team of surgeons. The antiviral-treated group (*n* = 42) showed overall survival rate of 88.1%, 79.1% and 71.2% at 1, 3, 5 years, respectively, while that of antiviral untreated cohort showed 76.5%, 47.5%, and 43.5% at 1, 3, 5 year, respectively.

Earlier, we reported an 8 year follow-up study of 15 patients with a single HCC (≤4 cm in diameter) 8. Following successful local tumor ablation, nine patients received antiviral therapy and six patients did not receive antiviral therapy due to unavailability. There was a significant difference in their survival; the median survival of the untreated and the treated was 12.5 and 60 months, respectively (*P* = 0.0001). All treated patients reached to undetectable HBV DNA within 10 months on antiviral therapy and maintained negative serum HBV DNA throughout. Recently, Wu et al. 10 in their study of Taiwan National Health Insurance data reported the results of a meta-analysis on the association between the nucleoside treated group and the untreated group as to the risk of HCC recurrence. The nucleoside analogues, LAM, ETV, and TLV, were the antivirals used. The treated group showed 20.9% of HCC recurrence rate (106/518) while the untreated cohort presented recurrence rate of 43.6% (1765/4051).

In this study, we expanded the inclusion criteria to a single tumor up to ≤7 cm in diameter and were able to include nine untreated controls and 15 treated. The treated group received the combination of LAM and TDF, LAM and ADV, or TLV and TDF. Within 10 months of antiviral therapy all treated subjects showed undetectable HBV DNA, and they maintained undetectable HBV DNA levels throughout with the HBV DNA assayed at six monthly intervals. Follow-up MRI after local tumor ablation was performed at 1 month and at three monthly intervals thereafter for the first 3 years and six monthly subsequently. Patients were followed by the same group of physicians at the clinic for over 12 years. Patients' compliance was excellent except one patient (Pt. no. 13) who after 1 year following TACE and antiviral therapy was lost in follow-up for a year. He returned 2 years later with recurrent HCC with the high HBV DNA level. He was restarted with antiviral and underwent more TACE but needed systemic chemotherapy. He died at 50 months after the index HCC diagnosis.

The carcinogenic process by sustained viremia with active viral replication has long been considered to be the result of the viral DNA insertion in or near proto-oncogenes, tumor suppressor genes or regulatory element of cellular DNA or the integration of viral DNA that leads to the production of the transactivator protein HBxAg and binding to P53 tumor suppressor genes. It appears that continuous viral replication is the key risk factor for recurrence and/or progression of hepatocarcinogenesis. It is interesting to speculate that even at the precancerous stage, elimination of the hepatocarcinogen and interruption of the final trigger may be able to prevent its progression to cancer. Therefore, successful control of HBV replication is of utmost importance in prevention of tumor recurrence and improvement of survival.

Similar observations have been made in hepatitis C related HCC by other investigators 15–19. Those who continued interferon therapy after tumor ablation had better survival than those who did not receive interferon therapy. This suggested that control of HCV replication was an important factor for improved survival of patients with HCV-related HCC.

Recently, ablative therapies have evolved with the development of more powerful devices which can deposit more energy into tumors and create a potentially greater zone of necrosis. While patients treated later in our clinic may have benefited from the improvement of available devices, it is important to consider that in all cases, our group achieved complete necrosis of the index tumor based on the follow-up imaging studies.

In this study, status of liver disease defined by Child-Pugh class was somewhat advanced for the untreated group (5A, 2B, 2C) than that for the treated group, (14A and 2B) although there was no significant difference (*P* = 0.169). In the untreated group, there were two patients who were Child-pugh class C and none in the treated group. Although not different statistically, their poorer liver function in the untreated may have partly contributed to their poorer survival. Nonetheless, the striking survival difference observed is far beyond this reasoning.

Furthermore, it is not known that while not receiving chemotherapy, this degree of difference of liver disease status would affect the development of new/recurrent HCC.

Our long-term study of over 12 years, albeit small in numbers, strongly indicates that even after developing HCC, continuous suppression of viral replication in CHB patients does reduce and prevent new/recurrent tumor and improves survival. Patients with a single small HCC, in addition to the local tumor ablation, should receive anti-HBV therapy. This novel treatment strategy offers an alternative to liver transplantation in patients with HBV-associated HCC.

## Conflict of Interest

None declared.
